# Ultra-processed food intake and diet carbon and water footprints: a national study in Brazil

**DOI:** 10.11606/s1518-8787.2022056004551

**Published:** 2022-02-16

**Authors:** Josefa Maria Fellegger Garzillo, Vanessa Fadanelli Schoenardie Poli, Fernanda Helena Marrocos Leite, Euridice Martinez Steele, Priscila Pereira Machado, Maria Laura da Costa Louzada, Renata Bertazzi Levy, Carlos Augusto Monteiro

**Affiliations:** I Universidade de São Paulo Núcleo de Pesquisas Epidemiológicas em Nutrição e Saúde São Paulo SP Brasil Universidade de São Paulo. Núcleo de Pesquisas Epidemiológicas em Nutrição e Saúde. São Paulo, SP, Brasil; II Deakin University Institute for Physical Activity and Nutrition Melbourne Austrália Deakin University. Institute for Physical Activity and Nutrition. Melbourne, Austrália; III Universidade de São Paulo Faculdade de Saúde Pública Departamento de Nutrição São Paulo SP Brasil Universidade de São Paulo. Faculdade de Saúde Pública. Departamento de Nutrição. São Paulo, SP, Brasil; IV Universidade de São Paulo Faculdade de Medicina Departamento de Medicina Preventiva São Paulo SP Brasil Universidade de São Paulo. Faculdade de Medicina. Departamento de Medicina Preventiva. São Paulo, SP, Brasil

**Keywords:** Diet, Ultra-Processed Food, Carbon Footprint, Water Footprint, Brazil

## Abstract

**OBJECTIVE:**

To study the association between ultra-processed food consumption and carbon and water footprints of the Brazilian diet.

**METHODS:**

Cross-sectional analysis on data collected in 2008–2009 on a probabilistic sample of the Brazilian population aged ≥ 10 years (n = 32,886). Individual food intake was assessed using two 24-hour food records, on non-consecutive days. The environmental impact of individual diets was calculated by multiplying the amount of each food by coefficients that quantify the atmospheric emissions of greenhouse gases in grams of carbon dioxide equivalent (carbon footprint) and freshwater use in liters (water footprint), both per gram or milliliter of food. The two coefficients consider the food life cycle ‘from farm to fork.’ Crude and adjusted linear regression models and tests for linear trends assessed the association between the ultra-processed food contribution to total energy intake (quintiles) and the diet carbon and water footprints. Potential confounders included age, sex, education, income, and region. Total energy intake was assessed as a potential mediation variable.

**RESULTS:**

In the crude models, the dietary contribution of ultra-processed foods was linearly associated with the carbon and water footprints of the Brazilian diet. After adjustment for potential confounders, the association remained significant only regarding the diet water footprint, which increased by 10.1% between the lowest and highest quintile of the contribution of ultra-processed foods. Additional adjustment for total energy intake eliminated this association indicating that the dietary contribution of ultra-processed foods increases the diet water footprint by increasing energy intake.

**CONCLUSIONS:**

The negative impact of ultra-processed foods on the diet water footprint, shown for the first time in this study, adds to the negative impacts of these foods, already demonstrated regarding dietary nutrient profiles and the risk for several chronic non-communicable diseases. This reinforces the recommendation to avoid ultra-processed foods made in the official Brazilian Dietary Guidelines and increasingly in dietary guidelines of other countries.

## INTRODUCTION

Food systems and supplies are transforming into a global industrial system, with corresponding changes in the production, distribution, and consumption of food^1^. As shown by time series on annual food sales available in 81 countries, including Brazil, the most conspicuous change has been the increase in the supply and consumption of ultra-processed foods^2^. In Brazil, national household surveys on food purchases have documented, since the 1980s, the displacement of fresh and minimally processed foods and their culinary preparations as dishes and meals by ultra-processed foods^3^.

Ultra-processed foods, as defined by the Nova classification^4^, are industrial formulations made mostly or entirely with substances extracted from foods, often chemically modified, and from additives, with little if any whole food added^5^. Among the characteristics that explain their increase in production and consumption are the relatively affordable price (due to low-cost ingredients), convenience (long duration and no need for cooking), hyper-palatability (due to large amounts of salt, sugar and/or fats, and classes of additives with cosmetic functions), and aggressive and sophisticated advertising (enabled by the high profitability of the products)^4^. Typical examples of ultra-processed products are soft drinks, packaged snacks, confectionery, reconstituted meat products, and frozen or shelf-stable ready meals^4^.

A systematic review and meta-analysis of findings from national dietary surveys carried out in many countries, including Brazil, shows that increases in the dietary share of ultra-processed foods are systematically associated with unbalanced dietary nutrient profiles, including higher free sugars and unhealthy fats and lower protein, fiber, and several vitamins and minerals^6^. Systematic reviews and meta-analyses of findings from long-term longitudinal studies, also carried out in many countries, including Brazil, show that increases in the dietary share of ultra-processed foods are associated with higher risk of obesity, diabetes, cardiovascular diseases, depression, and several other chronic non-communicable diseases, in addition to higher all-cause mortality^7^.

Given the evidence on the harmful health effects of ultra-processed foods and other factors, such as the negative impact that their production has on the environment, dietary guidelines in Brazil^10^, other Latin American countries^11^, France^12^, Israel^13^, and Malaysia^14^ state in their recommendations that the consumption of ultra-processed foods should be reduced or avoided.

The putative negative environmental aspects of production of ultra-processed foods include the reduced variety of species grown monoculturally on large tracts of land; the systematic use of fertilizers and pesticides on crops; the vast use of water; the many processes to formulate the final products; the transport over long distances; the waste generated by packaging; and the incentive to food consumption that exceeds physiological energy requirements^15,16^.

Empirical studies on the environmental impact of ultra-processed food production and consumption, although urgent, are still incipient^16^. A study carried out in the UK showed that industrially-prepared meals, probably ultra-processed, might have a greater environmental impact than equivalent homemade meals, in part due to storage in freezers and the quantity of package waste^17^. Another study showed that ‘discretionary foods,’ mostly ultra-processed, account already for more than a third of the carbon and water footprints of the Australian diet, a proportion expected to double by 2050^18^. In Brazilian metropolitan areas, the contribution of ultra-processed foods to the carbon and water footprints of the food baskets purchased by households has tripled in three decades (from 7.1% and 8.2% in 1987 to 20.4 % and 22.2% in 2018) following the increase in the consumption of those foods^19^.

In a previous study, based on a national dietary survey conducted in 2008–2009 in a probabilistic sample with more than 30,000 people, we estimated the carbon footprints of the Brazilian diet and its association with sociodemographic variables. The study identified larger footprints in diets consumed by men, by people between 20 and 49 years old, by people with higher income and higher education, and by residents in the North and Midwest regions of the country^20^. In our current study, we evaluate, for the first time in Brazil, and possibly in any country, the effect of the consumption of ultra-processed foods on the diet carbon and water footprints.

## METHODS

### Data Source

The data analyzed here are from the official national dietary survey conducted by the Brazilian Institute of Geography and Statistics (IBGE) between May 2008 and May 2009^21^.

The 2008–2009 IBGE dietary survey used a sampling plan by conglomerates created with the geographic and socioeconomic stratification of all census tracts of the country. Census tracts were drawn, in the first stage, and households, in the second stage. The final sample included 13,569 households. All residents aged 10 years or over were interviewed (n = 34,003).

Food intake was assessed using 24-hour food records, on non-consecutive days. Household members recorded all foods and culinary preparations with the amounts consumed and the form of preparation. Culinary preparations were separated into foods according to standard recipes^22^. The amounts of individual foods were transformed into grams or milliliters using the Brazilian Home Measures Table^23^ and converted into energy (kcal) based on the Brazilian Food Composition Table^24^.

Data on gender, date of birth, education level, family income, number of residents in the household, and region of residence, were obtained by the 2008–2009 IBGE survey using standard questionnaires.

### Data Analysis

The analyses used all the records of two days of food consumption (n = 32,886 or 96.8% of the total participants). Analyses have averaged the quantities of foods consumed in these two days.

The environmental footprints of the foods consumed were calculated by multiplying the average amount of each food by coefficients that quantify the atmospheric emissions of greenhouse gases, expressed in grams of carbon dioxide equivalent or gCO_2_eq (carbon footprint), and the use of fresh water, expressed in liters (water footprint), both per gram or milliliter of food.

The coefficients used were from the publication ‘Footprints of food and culinary preparations consumed in Brazil^22^. This provides the carbon and water footprints of all food items reported by the participants of the 2008–2009 IBGE survey. These coefficients consider the life cycle assessment of foods, including emissions from gas used in cooking (‘from farm to fork’). They were estimated from 569 primary sources of environmental food footprints, including scientific publications and reports prepared by industries on the environmental effects of their products. Arithmetical means were used when more than one value was available.

Using the Nova system^4^, all foods consumed by participants were classified into four groups consisting of unprocessed or minimally processed foods, processed culinary ingredients, processed foods, and ultra-processed foods.

Next, the dietary energy intake (kcal/person-day) and the dietary carbon and water footprints (gCO_2_eq/person-day and L/person-day, respectively) were estimated according to the four Nova food groups and subgroups (such as meat, plant oils, cheese, soft drinks). For each group and subgroup, the ratio between its percent contribution to the dietary carbon or water footprint and to the dietary energy intake was calculated. Thus, ratios equal to 1 identify food groups and subgroups with an environmental footprint (per unit of energy) identical to the whole diet. Ratios above 1 and below 1 identify food groups and subgroups with an environmental footprint (per unit of energy), respectively, higher and lower than the whole diet.

The effect that ultra-processed food consumption exerts on the carbon and water footprints of the Brazilian diet was then evaluated observing the diet footprints across five strata of the population corresponding to increasing quintiles of the contribution of ultra-processed foods to total energy intake. This was the same approach used by most of the studies evaluating the effect of consumption of ultra-processed foods on diet quality^6^ or disease risk^7^. Crude and adjusted linear regression models and linear trend tests were used to assess the association between quintiles of ultra-processed food dietary contribution and the dietary environmental footprints. Adjustments for potential confounders were made for sociodemographic variables that were associated with dietary footprints in the 2008–2009 IBGE survey^21^ (age, sex, education, income, and region of residence). Total energy intake was assessed as a potential mediation variable.

All analyses were performed using the software Stata/SE version 14.0 in the module survey, which considers the complex sample design of the 2008–2009 IBGE survey.

## RESULTS

Table 1 shows the distribution of the total dietary energy intake (1,905 kcal/person/day) and the total dietary carbon (4,488 gCO_2_eq/person-day) and water footprint (3,877 L/person/day) according to the Nova four food groups and corresponding subgroups.

**Table 1:** Distribution of dietary energy intake and the diet environmental footprints according to NOVA food groups and subgroups. Brazilian population aged 10 years or over, 2008–2009. (n = 32,886)

NOVA food groups and subgroups	Energy intake		Carbon footprint		Water footprint		Ratio	
			
	Kcal per person-day	% of total (a)	gCO_2_eq per person-day	% of total (b)	L per person-day	% of total (c)	(b)/(a)	(c)/(a)
Unprocessed or minimally processed foods	1,035.0	54.32	3,490.0	77.76	2,782.0	71.76	1.43	1.32
Rice	178.3	9.36	117.9	2.63	60.1	1.55	0.28	0.17
Meat^a^	170.9	8.97	2,583.0	57.55	1,786.0	46.07	6.42	5.14
Legumes	146.2	7.67	13.1	0.29	185.4	4.78	0.04	0.62
Poultry	125.4	6.58	275.9	6.15	373.7	9.64	0.93	1.46
Cereals other than rice^b^	119.2	6.26	27.7	0.62	49.8	1.28	0.10	0.21
Fruits	90.4	4.74	93.3	2.08	104.3	2.69	0.44	0.57
Roots and tubers	79.8	4.19	20.1	0.45	59.3	1.53	0.11	0.37
Milk	29.5	1.55	61.6	1.37	65.0	1.68	0.89	1.08
Fish	27.4	1.44	122.8	2.74	-	-	1.90	-
Eggs	21.8	1.14	52.3	1.17	47.8	1.23	1.02	1.08
Vegetables	10.2	0.54	18.5	0.41	28.5	0.74	0.77	1.37
Other^c^	35.5	1.86	9.2	0.20	16.0	0.41	0.11	0.22
Processed culinary ingredients	311.1	16.33	72.9	1.62	140.8	3.63	0.10	0.22
Plant oils	218.9	11.49	42.1	0.94	106.9	2.76	0.08	0.24
Table sugar	67.8	3.56	8.5	0.19	17.9	0.46	0.05	0.13
Butter and other animal fat	22.2	1.17	21.5	0.48	15.3	0.39	0.41	0.34
Other^d^	2.2	0.12	0.7	0.02	0.5	0.01	0.14	0.11
Processed foods	185.5	9.74	393.7	8.77	370.8	9.56	0.90	0.98
Fresh breads	121.3	6.37	27.4	0.61	73.0	1.88	0.10	0.30
Cheese	20.6	1.08	56.9	1.27	38.3	0.99	1.17	0.91
Processed meat	20.1	1.05	252.5	5.63	231.7	5.98	5.33	5.67
Beer and wine	16.0	0.84	45.6	1.02	15.7	0.40	1.21	0.48
Other	7.4	0.39	11.2	0.25	12.0	0.31	0.64	0.80
Ultra-processed foods	373.7	19.61	531.8	11.85	583.1	15.04	0.60	0.77
Ready meals^e^	80.8	4.24	148.1	3.30	104.0	2.68	0.78	0.63
Pastries	46.5	2.44	20.7	0.46	23.6	0.61	0.19	0.25
Confectionery^f^	39.3	2.06	30.1	0.67	119.7	3.09	0.33	1.50
Salty packaged snacks	37.7	1.98	16.6	0.37	12.7	0.33	0.19	0.17
Soft drinks	35.9	1.88	55.6	1.24	45.1	1.16	0.66	0.62
Sausages and other reconstituted meat products	33.0	1.73	168.1	3.75	171.9	4.43	2.16	2.56
Dairy drinks	30.1	1.58	46.9	1.04	46.1	1.19	0.66	0.75
Sauces	26.4	1.39	9.9	0.22	10.7	0.28	0.16	0.20
Mass-produced packaged breads	24.1	1.26	11.9	0.27	9.3	0.24	0.21	0.19
Other^g^	19.9	1.04	23.4	0.52	39.8	1.03	0.50	0.98
Total	1,905.3	100.00	4,488.4	100.00	3,876.7	100.00	1.00	1.00

^a^ Beef, pork, lamb.

^b^ Corn and wheat grains and flours, couscous, fresh or dry pasta.

^c^ Plain yoghurt, coffee, tea, oilseeds

^d^ Vinegar, baking powder.

^e^ Instant soups and noodles, fast food meals, frozen pizza, and pasta dishes.

^f^ Chocolates, candies and gums, cereal bars, desserts, ice cream.

^g^ Distilled alcoholic drinks and non-alcoholic drinks other than soft drinks.

The group of unprocessed or minimally processed foods contributed 54.32% of the total dietary energy intake and 77.76% and 71.76%, respectively, of the total dietary carbon and water footprints. The ratio between the contribution to footprints and the contribution to energy intake, referred to here as the footprint/intake ratio, was greater than 1 in this group, both for the carbon footprint (1.43) and for the water footprint (1.32), an environmental impact per unit of energy above that for the whole diet.

The group of processed culinary ingredients showed the opposite situation, corresponding to 16.33% of the energy intake and to only 1.62% of the carbon footprint and 3.63% of the water footprint of the diet. In this case, the footprint/intake ratio was well below 1 (0.10 for the carbon footprint and 0.22 for the water footprint), an environmental impact per unit of energy below that of the whole diet.

Usually consumed combined in dishes and meals, unprocessed or minimally processed foods and processed culinary ingredients have a joint environmental impact per unit of energy slightly higher than the whole diet: carbon footprint/intake ratio equal 1.13 and water footprint /intake ratio equal 1.07 (data not shown).

The group of processed foods contributed 9.74% of energy intake and 8.77% of the carbon footprint – footprint/intake ratio 0.90 – and 9.56% of the water footprint – footprint/intake ratio 0.98 – thus showing an environmental impact per unit of energy slightly lower than that of the whole diet.

The group of ultra-processed foods contributed 19.61% of energy intake and 11.85% of the carbon footprint – footprint/intake ratio 0.60 – and 15.04% of the water footprint – footprint/intake ratio 0.77 – also showing a lower environmental impact per unit of energy than that of the whole diet.

Within the Nova groups, the meat subgroups contributed disproportionately to the carbon and water footprints of diet, with footprint/intake ratios between 2.16 (for the carbon footprint of ultra-processed sausages and other reconstituted meat products) and 6.42 (for the carbon footprint of minimally processed meat). The lowest contribution of meat subgroups in the ultra-processed food group (1.73% in 19.61%, or 8.82% of the total energy intake from this group) and in the processed food group (10.78% of the total energy intake from this group) than in the unprocessed or minimally processed food group or in this group plus the group of processed culinary ingredients (16.51% or 12.70%, respectively) is also remarkable. This may explain why the environmental impact per unit of energy of the processed and the ultra-processed groups is lower than that of the whole diet.

Table 2 shows the total energy intake and the energy intake from ultra-processed foods across quintiles of their dietary contribution. This contribution ranged from 1.33% of the total energy intake in the lowest quintile to 44.64% in the highest quintile. The total energy intake increases linearly with the dietary contribution of ultra-processed foods: from 1,755.8 kcal/person-day in the lowest quintile to 2,075.3 kcal/person-day in the highest quintile.

**Table 2:** Dietary energy intake and energy intake from ultra-processed foods (UPF) across quintiles of their dietary contribution. Brazilian population aged 10 years or over, (2008–2009). (n = 32,886)

Quintiles of UPF contribution (% of total energy intake)	Mean energy intake (kcal per person-day)	Mean energy intake from UPF (kcal per person-day)	% of total energy intake from UPF	

			Average	Range
Q1	1,755.8	25.2	1.33	0–4.35
Q2	1,848.2	140.4	7.56	4.35–10.96
Q3	1,878.4	282.8	15.02	10.96–19.24
Q4	1,946.4	486.3	24.94	19.24–31.39
Q5	2,075.3	932.9	44.64	31.39–98.22
p	< 0.001	< 0.001	-	-

Table 3 shows results from regression models that evaluated the association between quintiles of the dietary contribution of ultra-processed foods and the diet carbon and water footprints.

**Table 3:** Diet environmental footprints across quintiles of the dietary contribution of ultra-processed foods (UPF). Brazilian population aged 10 years or over, (2008–2009). (n = 32,886)

Quintiles of UPF contribution (% of total energy intake)	Carbon footprint (gCO_2_eq/person-day)			Water footprint (L/person-day)		
	
	Crude	Adjusted^a^	Adjusted^b^	Crude	Adjusted^a^	Adjusted^b^
Q1	4,245	4,302	4,408	3,571	3,644	3,736
Q2	4,553	4,598	4,628	3,860	3,911	3,938
Q3	4,480	4,495	4,499	3,836	3,871	3,875
Q4	4,616	4,590	4,556	3,989	3,958	3,928
Q5	4,557	4,449	4,342	4,134	4,013	3,919
p value for linear trend	0.014	0.338	0.618	< 0.001	0.001	0.282

^a^ Adjusted for sex, age, education, income, and country’s region.

^b^ Adjusted additionally for total energy intake.

In the crude model, the dietary contribution of ultra-processed foods showed a linear significant association with the diet carbon and water footprints. In the model adjusted for potential confounders (gender, age, income, education, region of residence), only the association with water footprint remained significant. From the bottom quintile to the top quintile of the dietary contribution of ultra-processed foods, the diet water footprint increased by 10.1% (from 3,644 to 4,013 L/person-day).

In the model adjusted additionally for total energy intake, the association between the dietary contribution of ultra-processed foods and the diet water footprint was no longer significant. This indicates that the dietary contribution of ultra-processed foods increases the diet water footprint by increasing energy intake.

## DISCUSSION

Based on the analysis of data collected by two 24-hour food records in a probabilistic sample of the Brazilian population aged 10 years and over (n = 32,886), we found a significant linear association between the dietary contribution of the ultra-processed food group and the diet water footprint, even after adjusting for potential sociodemographic confounders. As far as we know, this is the first nationally representative study to show a negative environmental effect of consuming ultra-processed foods. The same linear association shown for the diet carbon footprint in the crude analysis was no longer significant after adjusting for the same sociodemographic variables.

Our results also showed that the additional adjustment for energy intake eliminated the association between the dietary contribution of ultra-processed foods and the diet water footprint, indicating the mediating role of total energy intake in that association.

The dietary contribution of ultra-processed foods was positively associated with the diet water footprint despite the lower water footprint of ultra-processed foods. This smaller footprint, apparently due to the smaller share of the meat subgroup in the ultra-processed group, failed to compensate for the higher total energy intake associated with the consumption of these foods. In any case, our results have shown that the association between the consumption of ultra-processed foods and diet environmental footprints cannot be predicted solely by comparing coefficients of the environmental impact of ultra-processed and non-ultra-processed foods, as it is sometimes done^25^.

Although we only evaluated two dimensions of the diet environmental impact in our study, the evidence that the consumption of ultra-processed foods increases the diet water footprint in Brazil, remaining neutral regarding the carbon footprint, adds to the evidence showing the negative effects of ultra-processed foods on the quality of the Brazilian diet^26^ and on the risk of several non-communicable diseases in the Brazilian population^27^. This reinforces the recommendation of the Brazilian Dietary Guidelines to avoid the consumption of ultra-processed foods^10^.

Given that the impact of the dietary contribution of ultra-processed food on diet environmental footprints depends on the footprints per energy of the ultra-processed and non-ultra-processed fractions of the diet, which, in turn, depend on the profile of ultra-processed and non-ultra-processed foods consumed by the population, the results of our study cannot be extrapolated to countries with considerably different dietary patterns to those of Brazil.

As indicated in our study, the proportion of meat products in the ultra-processed fraction of the diet is particularly important: when this proportion is lower than that observed in the non-ultra-processed fraction, as in the Brazilian diet, increases in the dietary share of ultra-processed foods are less likely to increase the diet environmental footprints. Another relevant factor is the relationship between the consumption of ultra-processed foods and the total energy intake: when this relationship is positive, as in the Brazilian diet and often observed in other countries’ diets^6^, increases in the dietary share of ultra-processed foods are likely to increase dietary environmental footprints. Thus, carrying out new studies on the effects of ultra-processed foods on dietary environmental footprints in countries with dietary patterns different from Brazil, in particular those with the highest consumption of ultra-processed foods, is important. Studies should also be carried out in countries that have already adopted the recommendation to limit or to avoid the consumption of ultra-processed foods in their dietary guidelines^10^, or are considering doing so.

A strength of our study is the assessment of the environmental impact of ultra-processed foods as actually consumed by the population. We were able to consider the mix of varieties of ultra-processed foods that are part of the diet, changes in this mix with variations in the dietary contribution of ultra-processed foods, and simultaneous changes in the varieties of non-ultra-processed foods. Other strengths are the representative sample of the population, the objective measurement of the participants’ diet based on two 24-hour food records, and the use of environmental impact coefficients that consider the entire life cycle of foods, from ‘farm to fork’.

A limitation is the assessment of only two environmental footprints of diet, not including, for example, impact on agrobiodiversity and the generation of solid waste from packaging. Also, the table of environmental impact coefficients used in the study, includes estimates from studies carried out in countries other than Brazil, and reports prepared by the industry, and not from independent studies.

## CONCLUSION

The negative impact of ultra-processed foods on the diet water footprint shown for the first time in this study adds to the negative impacts of these foods, already demonstrated regarding dietary nutrient profiles and the risk for several chronic non-communicable diseases. This reinforces the recommendation to avoid these foods made in the official Brazilian Dietary Guidelines and increasingly in the dietary guidelines of other countries.
